# Machine Learning Analysis for Quantitative Discrimination of Dried Blood Droplets

**DOI:** 10.1038/s41598-020-59847-x

**Published:** 2020-02-24

**Authors:** Lama Hamadeh, Samia Imran, Martin Bencsik, Graham R. Sharpe, Michael A. Johnson, David J. Fairhurst

**Affiliations:** 1 Department of Physics and Mathematics, School of Science and Technology, Nottingham Trent University, Nottingham, Clifton Campus NG11 8NS United Kingdom; 2 Exercise and Health Research Group, Sport, Health and Performance Enhancement (SHAPE) Research Centre, School of Science and Technology, Nottingham Trent University, Clifton Campus, NG11 8NS United Kingdom

**Keywords:** Data mining, Statistics, Design, synthesis and processing

## Abstract

One of the most interesting and everyday natural phenomenon is the formation of different patterns after the evaporation of liquid droplets on a solid surface. The analysis of dried patterns from blood droplets has recently gained a lot of attention, experimentally and theoretically, due to its potential application in diagnostic medicine and forensic science. This paper presents evidence that images of dried blood droplets have a signature revealing the exhaustion level of the person, and discloses an entirely novel approach to studying human dried blood droplet patterns. We took blood samples from 30 healthy young male volunteers before and after exhaustive exercise, which is well known to cause large changes to blood chemistry. We objectively and quantitatively analysed 1800 images of dried blood droplets, developing sophisticated image processing analysis routines and optimising a multivariate statistical machine learning algorithm. We looked for statistically relevant correlations between the patterns in the dried blood droplets and exercise-induced changes in blood chemistry. An analysis of the various measured physiological parameters was also investigated. We found that when our machine learning algorithm, which optimises a statistical model combining Principal Component Analysis (PCA) as an unsupervised learning method and Linear Discriminant Analysis (LDA) as a supervised learning method, is applied on the logarithmic power spectrum of the images, it can provide up to 95% prediction accuracy, in discriminating the physiological conditions, i.e., before or after physical exercise. This correlation is strongest when all ten images taken per volunteer per condition are averaged, rather than treated individually. Having demonstrated proof-of-principle, this method can be applied to identify diseases.

## Introduction

The generation of complex and varied patterns as a result of the liquid drying process is a common yet intriguing phenomenon in nature^1^. One of the first studies of drying droplets began with the publication in 1997 by Deegan *et al*.^2^ with an explanation of the formation of a ring-like structure commonly called a ‘coffee ring’ resulting from the evaporation of a droplet containing microparticles^3^. The reason for the formation of such a pattern is that the contact line, where the droplet meets the substrate, is pinned in place, due to the particles in the liquid. Consequently, liquid from the center of the droplet must flow outwards to replenish the liquid that evaporates at the rim bringing the particles with it^4^. The generic form of the coffee ring has been investigated and analysed by many researchers using different liquids including coffee^2,5,6^, nanofluids^7^, polymers^8,9^ and DNA^10^. Interestingly, in the past few decades, the analysis of patterns from dried droplets of biological fluids^11–13^ has gained a lot of attention due to applications in fields such as biomedical^14–16^ and forensic sciences^16,17^, with some reports of successful medical applications of the Litos test for analysing urine droplets^18^. Biological liquids, such as blood, are complex systems containing various components, including macromolecules and cells. When a droplet of blood is placed on a solid substrate to dry, a band of darker red forms at the periphery around the rim, much like the coffee ring. Inside this is a zone called the corona, which often contains clear radial cracks. The central zone is usually paler in colour and contains smaller, more randomly oriented cracks. There can be fractionation of the blood components between the regions of the droplet due to their size or mobility^19^. Among the many dried droplet patterns of biological liquids, those left by dried blood droplets have been investigated in some detail^20,21^. The overall dynamics of evaporation is often described by five separate stages^21,22^. Researchers have also investigated crack formation^19,21,23^, showing how the number of cracks increases with droplet diameter as well as the effect of substrate^24^, humidity^25,26^, evaporation rate^4^, surface roughness and wettability^16,27^ on the final pattern. Recently Smith *et al*.^28^ have shown that the drying mechanism of a blood droplet which is dropped onto a surface is influenced by the impact energy, which increases the droplet diameter and redistributes red blood cells within the droplet. Interestingly, blood samples have also consistently proven to be a key source for a wide variety of diagnostic and research purposes. Several studies showed that the patterns observed in dried blood droplets carry important information about the health status of humans and can be used for disease diagnosis^14,21,29^. Although similar drying processes occur during the drying of blood and colloidal suspensions, the variety, complexity and interactions between the various components of a blood droplet mean that much richer patterns are formed including radial and tangential cracks, colour variations, the formation of lobed fingers, fractionation of components and in some cases even spiral cracks. These various features must depend on the physical properties of the blood, which are in turn linked to the health condition of the person, for example to whether the person is healthy or suffers from anaemia or hyperlipidaemia^16^. Linking the observed patterns to the blood chemistry is a complex, multi-faceted challenge, but one that could lead to improvements in diagnosis of disease and other health conditions. Even though these methods have enabled researchers to identify distinguishing patterns in dried blood droplets, objective assessment systems are needed to remove subjective evaluation^20,30^. Recently, some technologies have been designed to overcome human subjectivity, such as acoustical-mechanical impedance (AMI)^31^. However there remains much scope for more stable and accurate technologies to be developed^15,20,32^. In this paper we develop sophisticated image processing routines and a highly-accurate machine learning algorithm to quantitatively and objectively discriminate the patterns from a large number of dried blood droplets, removing much of the subjectivity inherent in previous studies.

Increasingly sophisticated digital image processing algorithms have evolved to exploit the most informative features contained within an image. Over the years, methods have been developed to reduce variations in image quality^33^, for example, through spatial filtering^34^, edge detection^35^ and various types of interpolation; of which bi-linear interpolation^36^, cubic convolution^37^ and cubic spline interpolation^38^ are the most well-known and widely used. Moreover, in many applications, the primary interest is not in specific features of individual images, but rather in the image texture or the degree of regularity governing a pattern formed by multiple images. Such textures are most conveniently analysed not in the spatial domain but in the spatial frequency domain^39^. A very common method used in describing the distribution of an image’s pixel intensities as a function of space and frequency is the power spectrum^40,41^. Reducing or excluding the high-frequency components while preserving or choosing the low-frequency components helps in not only extracting and exhibiting the most useful information in an image but also reducing a large amount of noise.

The extraction of implicit, previously unknown, and potentially useful information from image data by using computer programs that automatically sift through a database, seeking regularities or patterns^42^ has recently become a popular and effective practice to discover real trends in raw data. One of the tools used to help with such extractions is machine learning^43–46^. In general terms, machine learning is a set of tools that allows users to teach computers how to perform tasks by providing examples of how they should be done. The increasing interest in machine learning methods, driven by the desire to discover trends or irregularities in huge databases, has led to many successful applications of machine learning in various domains, taking advantage of the development of robust and efficient algorithms to process data and the falling cost of computational power^47–50^. Particularly, machine learning algorithms have been used and developed extensively in the domain of image analysis^51,52^. Blood images are governed by a large number of physiological and environmental variables some of which might be correlated. These correlations bring about a redundancy in the information that can be gathered by the dataset of the images. One approach to coping with redundancy is to reduce the dimensionality of the problem by combining data from correlated features. The success of reducing dimensionality is enabled by (i) significant performance gains in computational speed and memory and (ii) the generation of physically interpretable spatial modes that are linked to the underlying physics^53^. Linear combinations between variables are particularly attractive because they are simple to compute and analytically tractable. One of the most ubiquitous methods in dimensionality reduction is principal component analysis (PCA)^54^. PCA is mathematically defined as an orthogonal linear transformation that reconstructs the data to a new coordinate system such that the greatest variance by some projection of the data comes to lie on the first coordinate (called the first principal component), the second greatest variance on the second coordinate, and so on^55^. Furthermore, classification methods are often performed in a tailored low dimensional basis extracted as hierarchical features of the data^56^. Linear discriminant analysis (LDA)^57,58^ is a supervised learning method that is commonly applied in conjunction with PCA for discrimination tasks. LDA searches for those vectors in the underlying space that best discriminate between classes. More formally, given a number of independent features derived from the data, LDA creates a linear combination of those that yield the largest mean differences between the desired classes^59^. Indeed, PCA-LDA is one of the classic approaches used to introduce machine learning methodologies^54^. PCA and LDA have both been used extensively for biological data discrimination purposes^60,61^ and for image processing^56,62^.

In this work, we quantitatively and objectively analyse the power spectrum of a dataset of over one thousand images of dried blood droplets. The blood is taken from thirty healthy male volunteers at five time points: before an exhaustive cycling exercise, at peak exertion, and at two, four and six minutes into the post-exercise recovery. Our overall goal is to develop an image processing method to identify and extract the most distinguishing features in the images and apply an advanced multivariate statistical machine learning algorithm to quantitatively and objectively discriminate between the images. This will allow us to identify, from images of dried blood droplets, the physiological states of the cyclists; whether the person is at rest or has performed physical exercise.

This paper is organised as follows. First we present the results obtained from optimising our machine learning statistical model when applied to our image-processed dried blood droplet dataset. Next, we discuss these results and the potential use for current and future work. Finally we explain the methods followed in this work.

## Results

### Exercise responses and blood chemistry analysis

**Figure 1:**
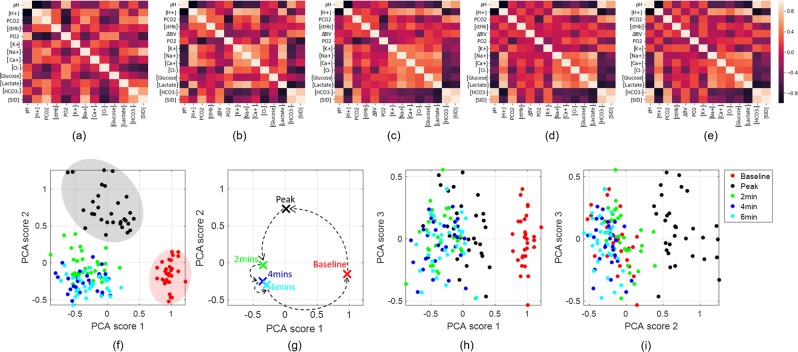
Statistical analysis of the measured blood chemistry properties. Correlation matrices are calculated for the 14 properties measured at (**a**) baseline, (**b**) peak exercise, (**c**) after 2 minutes exercise, (**d**) after 4 minutes exercise and (**e**) after 6 minutes exercise. The first three scores of the unsupervised dimensionality reduction and clustering method, principal component analysis, are shown in separate 2D plots (**f,h,i**) for the chemical properties: [H^+^], PCO_2_, [Hb], *Δ*BV, PO_2_, [K^+^], [Na^+^], [Ca^2+^], [Cl^−^], [Glucose], [Lactate^−^] and $[{{\rm{HCO}}}_{3}^{-}]$, discarding pH and [SID] as they are related to the other quantities. The best discrimination between all five blood conditions is revealed along the first principal component score shown in (**f**). The centroid’s behaviour related to the conditions clusters is shown in (**g**) which strongly suggests that the blood condition, from the chemistry point of view, is returnings towards baseline after 6 minutes of exercise.

The gas exchange threshold during the cycling exercise test was 2.81 ± 0.66 L ⋅ min^−1^ (250 ± 62 W). Peak cycling power, maximum oxygen uptake and maximum heart rate were 335 ± 64 W, 3.63 ± 0.73 L ⋅ min^−1^ (47.7 ± 8.9 mL ⋅ kg^−1^ ⋅ min^−1^), and 184 ± 12 bpm, respectively. For all blood parameters, data were analysed in GraphPad Prism (V7.05) using repeated measures analysis of variance and Tukey’s multiple comparison post-hoc test. The blood parameters measured in this study included pH, partial pressures of carbon dioxide and oxygen (PCO_2_, PO_2_), and concentrations of K^+^, Na^+^, Ca^2+^, lactate (La^−^), Cl^−^, ${{\rm{HCO}}}_{3}^{-}$, glucose and haemoglobin (Hb). Subsequently, $\left[{{\rm{H}}}^{+}\right]$, the strong ion difference ($\left[{\rm{SID}}\right]$) and changes in blood volume from rest (*ΔBV*) were calculated. In Fig. 1, we present the correlation matrices between all these chemical properties for all five measurement points. It can be seen that a remarkable correlation, revealed by instances of black or pale pink pixels, between some of these properties occurs when changing the condition of the blood. For instance, for blood taken at rest, pH and PCO_2_ show a strong negative correlation illustrated by the third dark square in the top row in Fig. 1a. Exercise reduces this correlation until the final blood sample taken after 6-min recovery Fig. 1e, as shown by the red square which indicates a much lower correlation. These features would be expected based on physicochemical principles of blood acid-base balance^63^. Specifically, changes in pH (or $\left[{{\rm{H}}}^{+}\right]$) and $\left[{{\rm{HCO}}}_{3}^{-}\right]$ are determined by changes in $\left[{\rm{SID}}\right]$, the total concentration of weak acids in the blood, and PCO_2_^63^. At rest, between-participant differences in blood pH are mainly determined by differences in PCO_2_ since $\left[{\rm{SID}}\right]$ and the total concentration of weak acids are usually kept within narrow limits both within- and between-participants. During exercise, however, $\left[{\rm{SID}}\right]$ falls due mainly to an increase in $\left[{{\rm{La}}}^{-}\right]$, whereas PCO_2_ may change little from rest and actually decline in recovery (as shown in Table 1). Thus, the fall in blood pH during and after maximal exercise is primarily explained by reductions in $\left[{\rm{SID}}\right]$, with increases in the total concentration of weak acids making an additional, albeit smaller, contribution. These principles are therefore consistent with the reduced correlation between pH and PCO_2_ as the experiment progressed.

**Table 1:** Measured blood variables at rest, at the end of exercise, and at 2-, 4- and 6-min recovery. Values are mean ± standard deviation. For all variables there was a significant (*P* < 0.001) change over the duration of the experiment. Significant changes from the preceding value are indicated with ^*^ for *P* < 0.05 and ** for *P* < 0.001.

	Rest	End of Exercise	Recovery (min)		
			2	4	6
$\left[{{\rm{La}}}^{-}\right]\left({\rm{mmol}}\cdot {L}^{-1}\right)$	1.1 ± 0.4	10.8 ± 3.1**	15.2 ± 2.2**	15.6 ± 2.2	15.3 ± 2.2
$\left[{{\rm{Na}}}^{+}\right]\left({\rm{mmol}}\cdot {L}^{-1}\right)$	141 ± 1	148 ± 2**	146 ± 1**	144 ± 1**	143 ± 1**
$\left[{{\rm{K}}}^{+}\right]\left({\rm{mmol}}\cdot {L}^{-1}\right)$	4.0 ± 0.2	5.4 ± 0.6**	4.1 ± 0.2**	3.8 ± 0.2**	3.8 ± 0.2
$\left[{{\rm{Ca}}}^{+2}\right]\left({\rm{mmol}}.{{\rm{L}}}^{-1}\right)$	1.23 ± 0.03	1.31 ± 0.04**	1.28 ± 0.03**	1.26 ± 0.03**	1.24 ± 0.03
$\left[{{\rm{Cl}}}^{-}\right]\left({\rm{mmol}}\cdot {L}^{-1}\right)$	105 ± 1	108 ± 2**	107 ± 2**	106 ± 1	106 ± 1
$\left[{\rm{SID}}\right]\left({\rm{mmol}}\cdot {L}^{-1}\right)$	40 ± 2	34 ± 3**	28 ± 2**	27 ± 2^*^	26 ± 2
${{\rm{PCO}}}_{2}\left({\rm{mmHg}}\right)$	44.9 ± 4.1	52.8 ± 9.9**	36.3 ± 3.9**	33.8 ± 3.2	33.2 ± 2.7
$\left[{{\rm{H}}}^{+}\right]\left({\rm{nmol}}\cdot {L}^{-1}\right)$	40.8 ± 2.2	61.8 ± 8.6**	63.8 ± 7.0	65.4 ± 7.2	65.1 ± 7.1
$\left[{{\rm{HCO}}}_{3}^{-}\right]\left({\rm{mmol}}\cdot {L}^{-1}\right)$	25.7 ± 1.1	18.4 ± 2.1**	14.8 ± 1.5**	14.0 ± 1. 4^*^	13.9 ± 1.5
$\left[{\rm{Hb}}\right]\left({\rm{g}}\cdot {{\rm{dL}}}^{-1}\right)$	15.4 ± 1.1	16.7 ± 1.0**	16.7 ± 1.0	16.6 ± 1.0	16.4 ± 1.0

We then use PCA to uncover linear combinations of these fourteen values which vary between conditions and will ultimately not only significantly reduce the dimensionality of the blood data but also show a possible clustering between the blood conditions. Fig. 1f,h,i show the relationship between pairs from the first three PCA scores demonstrating that, not only have we reduced the dimensionality of the system to a feature subspace, but the first and second PCA scores also give excellent unsupervised clustering of the five conditions as baseline and peak data points fall into two non-overlapping clusters (shaded), with both scores contributing equally to distinguish the physiological states. On the other hand, although there is an overlap between all the three sets of recovery data points, they are noticeably separated from baseline clusters using the first PCA score and from the peak cluster using the second PCA score. The third PCA score does not offer any additional distinction, as shown in Fig. 1h,i. In addition, the behaviour of the centroids of each cluster, indicated by the crosses on Fig. 1g, shows that from a chemical point of view, the condition of the blood is returning towards the baseline status in a loop-like manner. This is manifested by the combination of the first pair of PCA scores, with the first one carrying more information than the second one. Numerically, had we continued to take samples longer into the recovery period, the centroids would eventually return to the baseline position.

### Power spectrum discrimination

**Figure 2:**
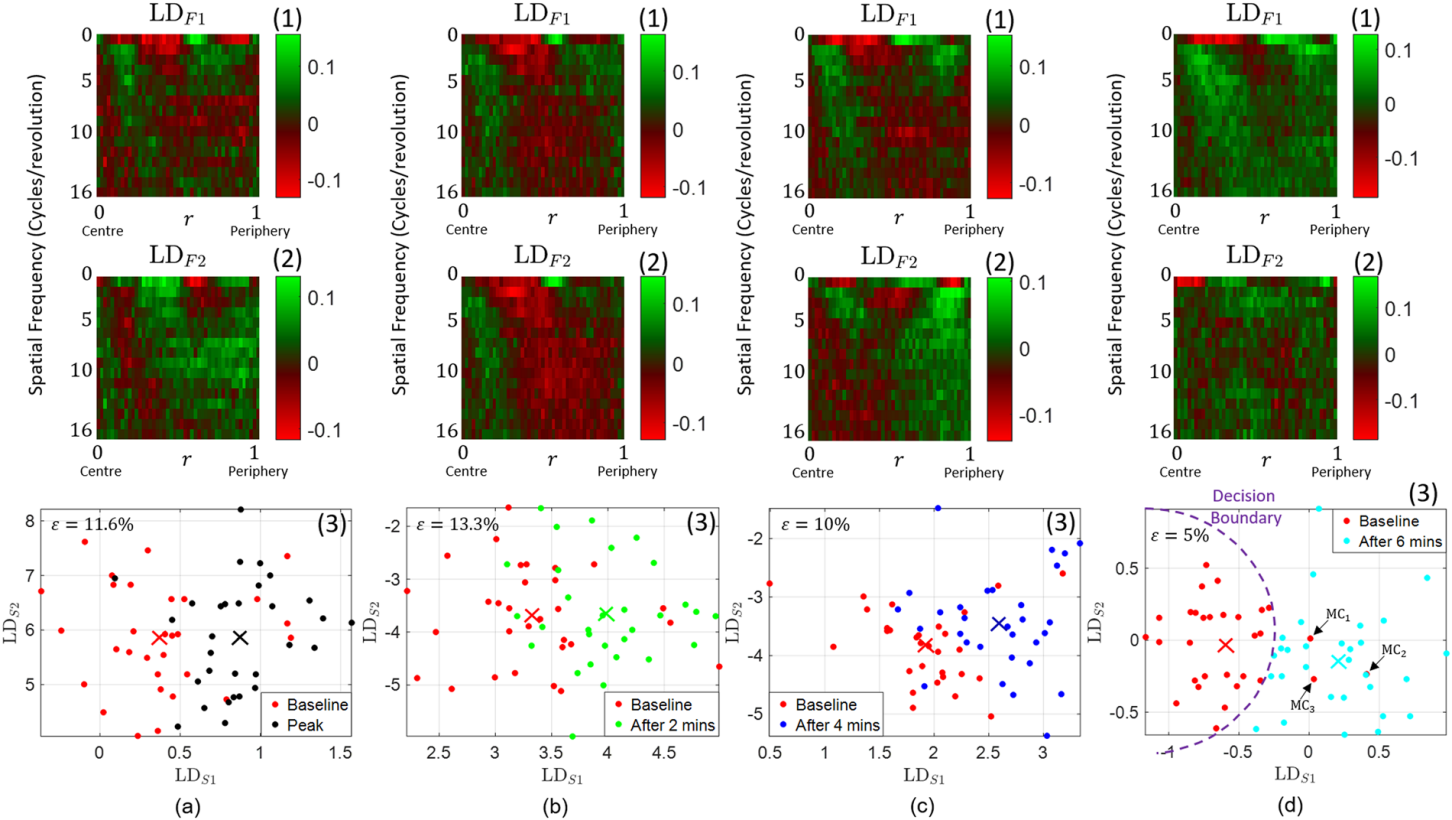
The discrimination outcome of the optimised machine learning algorithm to discriminate the logarithmic power spectrum of two blood conditions: (**a**) baseline against peak, (**b**) baseline against after 2 mins, (**c**) baseline against after 4 mins and (**d**) baseline against after 6 mins. The lowest error rate has been obtained when discriminating baseline and after 6 mins with *e* = 5% shown in the scatter plot (d,3) that illustrates the relationship between the first two LDA scores, i.e., LD_*s*1_ and LD_*s*2_. Here MC_*i*_, where *i* = 1, 2, 3, refers to the misclassified baseline points. Figures (d,1) and (d,2) represent the first and second LDA functions, respectively, where each element of each row (frequency) corresponds to the average pixel intensity at a given radius. The spatial frequency of the linear discriminant functions is measured in units of cycles per revolution.

We developed an optimised machine learning algorithm that successfully discriminates the logarithmic power spectrum of the volunteer-averaged images. The feature extraction is carefully described in the Methods section later on. We first perform a dimensionality reduction using principal component analysis (PCA) followed by a supervised discrimination method known as linear discriminant analysis (LDA) which linearly combines the PCA scores to optimise the discrimination between the images of two specific different conditions under scrutiny. For each condition per participant, ten to twelve multiple images were acquired. Fig. 2 shows the outcome of the discrimination process between baseline and the other conditions over the average of all the images per participant per condition. It can be seen in Fig. 2d that the error in discrimination is lowest, 5%, between the baseline images and those taken after 6 minutes of recovery. Both classes are clearly discriminated mostly along the first linear discriminant score, LD_*S*1_, as shown in Fig. 2d,3 In addition, Fig. 2d,1 shows the first linear discriminant function which exhibits the behaviour of the spatial frequency as a function of radial information. It can be noticed, by a simple projection, that a clear pattern indicates rich discriminating information situated close to the droplet’s centre and near its periphery. The bright green and red patches shown in the linear discriminant function provide important fingerprints that can be used to discriminate new images. Although the second linear discriminant score, LD_*S*2_, shows a poor projection axis for separating the centroids, in Fig. 2d,2, it reduces misclassification when using an appropriate decision boundary such as the circle exemplified in Fig. 2d,3. These two functions are able to classify the "baseline” and "after 6 mins” conditions of unknown blood droplet images with a high accuracy of *η* = 95%.

**Figure 3:**
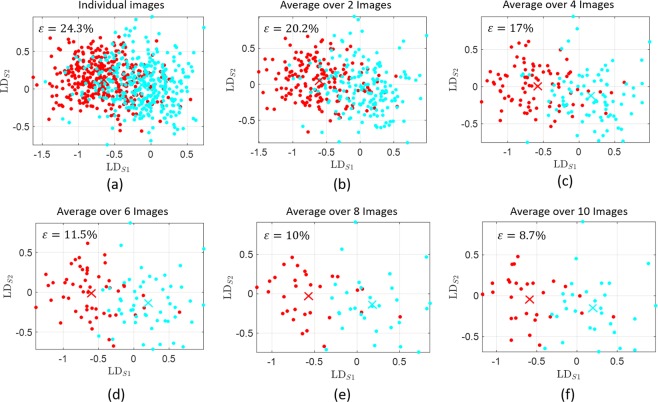
The effect of averaging separate images from the same volunteer and the same physiological state, on the final discrimination outcome. In (**a**) individual images with no averaging are used for the discrimination process. The accuracy of this approach is 75.7%. The accuracy increases noticeably with increasing the number of averaging images in (**b**–**f**). As shown previously in Fig. 2d,3, all images (usually ten to twelve) taken per volunteer per condition are averaged and the accuracy has considerably increased to 95%. An optimised training exercise was undertaken for each separate case.

We then used the comparison between droplets taken at baseline and after 6 minutes recovery, to investigate the effect of changing the number of averaging images on the final error rate of the statistical model. We find that averaging over all the images per participant per condition gives the best discrimination. Passing the entire database of individual images to our predictive model lowers its accuracy to nearly 20% as demonstrated in Fig. 3a. This error rate is gradually lowered by averaging more images per participant per condition until it reaches the best discrimination accuracy of 95% illustrated in Fig. 2d,3. This suggests that using multiple images is necessary, as it averages out the random variations between all droplets, highlighting the true differences due to blood differences.

**Figure 4:**
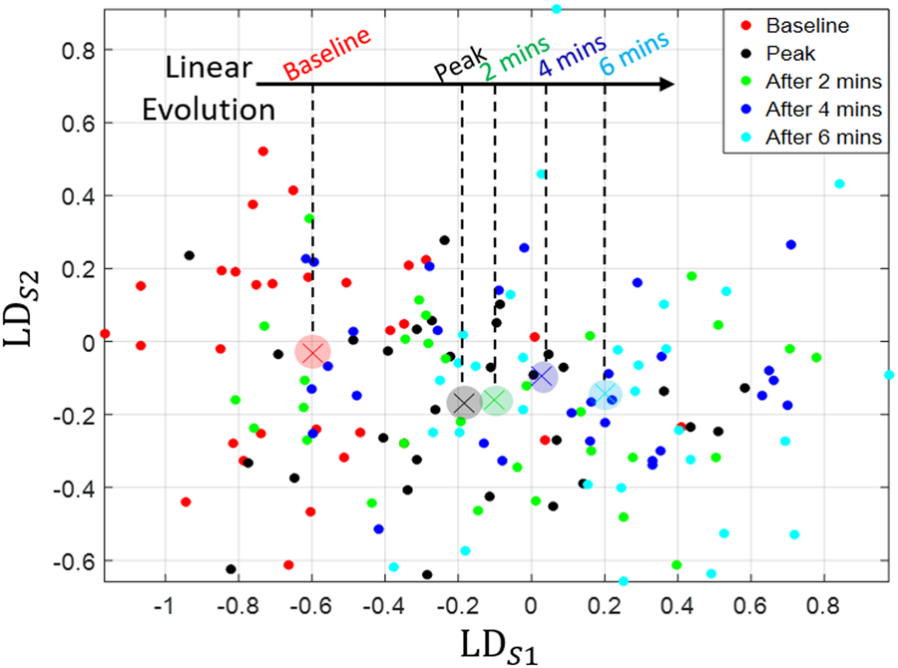
The trajectory of the centroid of the blood condition clusters, with images averaged over each person. The individual volunteer’s images, projected onto our DF space, are also shown, to disclose the inter-subject variability.

Additionally, the behaviour of the centroid of each condition class, indicated by the crosses on Fig. 4, is also analysed. This shows, in clear contrast with the behaviour seen in Fig. 1g, that the centroid of each condition’s cluster moves monotonously and linearly only along the LD_*S*1_ axis over time. Unlike the blood chemistry centroid trajectory presented in Fig. 1g, here we do not see the blood droplet patterns returning towards their original baseline state.

## Discussion

Some researchers have used drying blood droplets to diagnose disease. One paper^64^ reported attempts to use droplets of blood serum to diagnose cancer as "statistically unreliable”, yet the authors were able to qualitatively visualise the profound changes in the phase transition and the phase state of blood plasma proteins in patients with metastatic cancer. However, others have had more success. In ref. ^31^ images are presented from a blood serum with a range of diseases, including cancers and hepatitis. These droplets are distinguished statistically using the “shape index” of the time evolution of the acousto-mechanical impedance of the droplets, measured using a quartz resonator. The shape index shows good specificity and sensitivity to the various conditions. Further studies^65^ have used manual identification of morphological features in dried droplets (which the authors call ‘facias’) of blood serum, tears and synovial fluid and present statistically significant correlations between features and conditions. A similar approach has found quantitative discrimination for identifying hepatitis^66^ in blood serum, and shown qualitative differences due to anaemia and hyperlipidemia^21^.

The presence of various proteins in blood serum is shown to qualitatively affect the microstructure of the dried film^67^ with some success in a quantitative analysis by manually categorising the orientation of cracks. A more quantitative approach, in which crack angle and plaque sizes are measured, shows a convincing correlation with relative humidity^68^. An alternative automated approach^69^ calculating a “distance” between images of uninfected blood droplets and those with either tuberculsosis or anemia shows some promise.

A recent study^70^ is the first to probe the physical mechanisms linking measured blood properties to the observed patterns in whole blood droplets. The authors find that a normalised capillary pressure (which depends on blood viscosity, mean corpuscular volume and haematocrit), controls the properties of the cracks that form in healthy blood compared to that with neonatal jaundice and thalassamia. They also observed that droplet drying times vary with disease conditions.

A recent review article^71^ discusses spectroscopy of droplets, and makes conclusions regarding the optimal conditions, suggesting using “a diluted aliquot of serum (1 *μ*L) spotted on a smooth, flat and homogeneous surface, at an elevated temperature or high humidity in order to result in a more uniformly spread sample, with a faster drying time”.

Our approach differs from other techniques as we do not impose a discrimination based on human observations of distinguishing features. Instead, we employ a machine-learning optimisation technique which discovers the statistically most significant measure to classify and identify the different conditions. Our approach has the potential for objectively diagnosing medical conditions using fully automated image processing tools.

Our findings show for the first time how quantitative and objective image analysis of patterns left by dried blood droplets can succeed in detecting dominant features and optimally discriminating different physiological conditions, with an error of 5%. This approach has great potential in providing a new, rapid and reliable method for not only discriminating healthy blood conditions, but also in disease detection.

The images have been analysed and prepared before passing them on to the machine learning algorithm for discrimination. This analysis consists of several steps: applying interpolation to homogenise the spatial extent of any structure of each image; removing cracks and discontinuities found to be appearing inconsistently; extracting the most useful information that might be implicit in the image function; converting to polar coordinates; and finally calculating the power spectrum of the angular variations, removing any dependency on droplet absolute orientation.

Our machine learning algorithm is performed first using principal component analysis, as an unsupervised dimensionality reduction method, followed by linear discriminant analysis, as a supervised classification method. These two methods are very common for discrimination purposes. However, our novel contribution lies in developing a time-saving optimisation process to enhance the algorithm’s training capability and improve its prediction outcome. This process is applicable to large datasets since it does not search within all possible combinations of participants for the ideal and lowest error rate possibilities. Instead, it systematically ’selects’ the training data that we would like the iterative process to be applied to. This significantly lowers the computation time and successfully trains the algorithm. The optimised algorithm yielded a high discrimination accuracy of 95%, typically 10% higher than when using a standard analysis. Note that the numerical search outputs discriminating features that were determined using up to 50% of the PC scores, thereby using details that cannot necessarily be identified by the naked eye. In fact, only averaging numerous images belonging to specific categories allows the user to discriminate them by visual inspection, substantiating the need for numerical discrimination.

Our work highlights the importance of averaging over a sufficient number of droplet images for each individual as this improves the final discrimination accuracy. It is also interesting to note that the centroids representing the blood images move in a linear direction, and have not started to show any sign of returning to baseline after 6 minutes of recovery. In contrast, the blood chemistry centroids seem to have almost recovered, by that time. A summary of statistical analyses of the blood chemical properties is given in Table 1 and clearly shows that there is no variable that continues to move away from the resting value. For future studies, we would recommend allowing recovery to continue for much longer after exercise, possibly up to one hour. This observation also suggests that some other property of the blood is affecting the droplet patterns than any of the parameters that were measured.

## Methods

**Figure 5:**
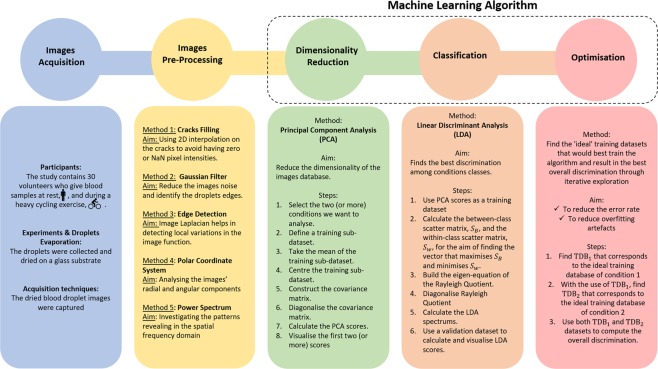
Work methodology. The study begins by building the database of the dried blood images taken from 30 healthy volunteers before and after a cycling exercise. These images are then pre-processed individually using different image analysis techniques; cracks filling, Gaussian filter, edge detection, polar coordinate and power spectrum. The database of the logarithmic power spectrum of the images are later used to be passed on to our machine learning algorithm. This algorithm starts with reducing the dimensionality of the image database using principal component analysis. Using the resulting feature space, i.e., the subspace that contains the highest varying principal components, we can move on to the classification step where we use linear discriminant analysis to find the best line that best separates the conditions of the images. The performance of this algorithm is further enhanced by applying an optimising iterative search to select the ‘ideal’ few participants who would best train the algorithm and result in the lowest overall error rate possible.

In this section, we present and explain the methods adopted in this work with the aim of quantitatively and objectively discriminating the conditions of the dried blood droplet images. The experimental protocols (exercise test and blood sampling) were approved by the Nottingham Trent University Human Ethical Review Committee. All methods were performed in accordance with the relevant guidelines and regulations of the standards set by the Declaration of Helsinki. There are no competing interests associated with this work. The main stages followed in this work can also be seen in Fig. 5.

### Participants, exercise protocol and blood sampling

Thirty healthy, recreationally active, non-smoking, males (age: 24 ± 6.9 years; height: 178 ± 5.5 cm; body mass: 76 ± 9 kg) provided written informed consent to take part in the study. Participants performed a maximal incremental cycling ramp test on an electromagnetically braked cycle ergometer (Excalibur Sport; Lode, Groningen, the Netherlands). Participants performed 3 minutes of unloaded cycling followed by an incremental ramp protocol (30 W ⋅ min^−1^), at their preferred cadence, until the limit of tolerance or task failure (cadence below 60 rpm). Thereafter, participants remained seated on the cycle ergometer for 6 minutes. During exercise participants wore a facemask (model 7940; Hans Rudolph, Kansas City, MO) connected to a flow sensor (ZAN variable orifice pneumotach; Nspire Health, Oberthulba, Germany) that was calibrated using a 3-L syringe. Gas concentrations were measured using fast responding laser diode absorption spectroscopy sensors which were calibrated using gases of known concentration (5% CO_2_, 15% O_2_, balance N_2_), and ventilatory and pulmonary gas exchange variables were determined breath-by-breath (ZAN 600USB; Nspire Healh). The gas exchange threshold was determined using the V-slope method^72^ and the maximum oxygen uptake was taken as the highest 10 second mean value. Heart rate was measured using short-range telemetry (Polar S160, Polar, Kempele, Finland). Arterialised venous blood (2 mL) was drawn from a heated (using an infra-red lamp) dorsal hand vein using an indwelling 21-G cannula and a syringe containing dry electrolyte-balanced heparin (*safe*PICO, Radiometer, Copenhagen, Denmark). Blood samples were taken at rest, at the end of exercise, and after 2-, 4- and 6-min recovery. Blood was analysed immediately (ABL90 FLEX; Radiometer) for pH, PCO_2_, $\left[{{\rm{HCO}}}_{3}^{-}\right]$, $\left[{{\rm{K}}}^{+}\right]$, $\left[{{\rm{Na}}}^{+}\right]$, $\left[{{\rm{Ca}}}^{2+}\right]$, $\left[{{\rm{La}}}^{-}\right]$, $\left[{{\rm{Cl}}}^{-}\right]$, $\left[{\rm{glucose}}\right]$, and $\left[{\rm{Hb}}\right]$. The remaining blood was transferred into a heparin-coated Eppendorf tube for subsequent blood droplet analysis. The hydrogen ion concentration ($\left[{{\rm{H}}}^{+}\right]$) was calculated from the measured pH value. The strong ion difference ($\left[{\rm{SID}}\right]$) was calculated as the difference between the sum of the strong cations and the strong anions: $\left[{\rm{SID}}\right]=(\left[{{\rm{Na}}}^{+}\right]+\left[{{\rm{K}}}^{+}\right]+\left[{{\rm{Ca}}}^{2+}\right])-\left(\left[{{\rm{Cl}}}^{-}\right]+\left[{{\rm{La}}}^{-}\right]\right)$^63^. We calculated *ΔBV* from resting levels using changes in $\left[{\rm{Hb}}\right]$^73^.

### Droplet preparation and evaporation

Significant care was required to deposit the droplets repeatably. A micro-pipette was used to produce a 10 *μ*L droplet at the tip of the pipette, small enough so that gravity did not cause it to fall. The pipette was slowly lowered until the droplet came into contact with the glass slide, when it would transfer to the substrate with minimal impact velocity. The glass slides measured 76.2 mm × 25.4 mm and were first cleaned with an air duster. For each blood sample, at least 12 droplets were pipetted on each of two slides in two rows of six. The spacing between droplets was around four times the radius, reducing any possible effects of crowding. Immediately after pipetting, the droplets were very mobile so the slides were left for between 10 to 12 minutes. After this time, the very edge of the droplet had dried sufficiently to pin the liquid to the glass, enabling the slides to be moved without risk of disturbing their circular shape. The slides were then transferred to an air-tight Bel-Art transparent desiccator with dimensions 337mm wide by 254mm deep by 216mm tall, and left there overnight.

The chamber was fitted with a mechanical hygrometer to measure the relative humidity inside the chamber, but we also used a humidity probe to monitor temperature and humidity during drying. The chamber had a movable bottom shelf which contained regular holes allowing for air exchange. Beneath the shelf we placed a Petri dish containing a supersaturated solution of NaCl salt, which fixed the relative humidity in the chamber in the range 70% to 75%^74^. At this humidity, the evaporation rate was slow, taking around 24 hours to fully dry, and reducing the influence of neighbours. The chamber had a capacity to accommodate 16 to 18 glass slides.

### Image acquisition

Droplet images were recorded using a CASIO EX-Z1000 digital camera in HDR super-macro mode with fixed magnification, with resolution 150 px/mm mounted 9cm above a white flat backlight (The Imaging Source DCL/BK.WI 5070/EU). Reflections from other lights in the room were eliminated using screens. As the dried deposits had a tendency to flake or peel, the slides were carefully transferred from the drying chamber and placed directly on top of the backlight. Images were cropped manually to contain one complete droplet per image. The dataset of the dried blood images can be accessed via 10.6084/m9.figshare.11373948.v1.

### Image pre-processing

**Figure 6:**
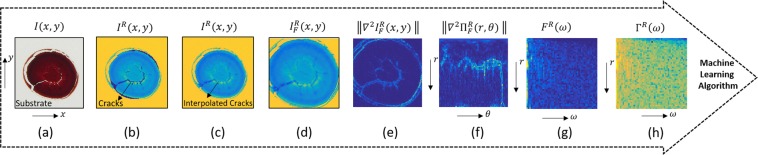
Image pre-processing methods where (**a**) shows the raw blood image, (**b**) represents the red channel of the image, (**c**) illustrates the 2D interpolated cracks, (**d**) shows a 2D Gaussian filtered image, (**e**) shows the Laplacian of the image function, (**f**) is the polar coordinate version of the image, (**g**) is the 1D power spectrum of the image previously shown and (**h**) is the logarithmic power spectrum.

After recording, documenting and cropping the raw blood images, image processing routines must be implemented before quantitatively analysing the data. Here, we present all the image pre-processing techniques that we have applied on our blood raw images for the aim of (i) reducing their noise, (ii) enhancing their quality and (iii) extracting the most important information to fully prepare them for the classification process. This improves the accuracy of the subsequent statistical analysis and makes the discrimination process well-trained. Fig. 6 illustrates the image analysis techniques applied on each image of our database before proceeding to the discrimination algorithm.

- Crack filling: Cracks often appear in dried droplet patterns and can be wavy, straight, arched, spiral, circular, and three-armed cracks^75– 81^. Significant work has been done on explaining the reasons behind crack-formation and the parameters affecting their formation in dried droplets, and specifically in blood droplets^4,20,21^. Cracks appear as white pixels in the image with maximum pixel intensity, and as others have reported “cracked surfaces across a drop can generate spectral distortions often in the form of baseline discrepancies”^71^. In our situation, the cracks deteriorate the study quality as they appear inconsistently in our data set. Therefore, we decided to automatically ‘airbrush’ the cracks by performing a 2D interpolation reducing this discontinuity. Since cracks usually have low luminance and due to the fact that most useful information in the blood droplets images are concentrated in the red channel, our crack detection criterion focuses on the red-channel of the images with a condition that the other two colour channels; blue and green, are above a certain value, i.e., ${{\boldsymbol{I}}}^{R}(x,y)\left[{\rm{if}}:{{\boldsymbol{I}}}^{B,G}(x,y) > \alpha \right]$, where ***I***^*R*^(*x*, *y*) and ***I***^*B*,*G*^(*x*, *y*) are the image matrix functions in the Cartesian coordinate system for the red and blue-green colour channels, respectively, and *α* is the value that best reveals the cracks, as shown in Fig. 6b. This enhances the clarity of the cracks where presented in an image. Once we identify the position of the cracks, a 2D linear scattered interpolant method is applied to interpolate their values, as shown in Fig. 6c.
- Gaussian filter: To consistently detect the boundaries of the blood droplet, ignoring marks on the substrate, edge detection must be preceded by the use of a 2D Gaussian filtering, also smoothing out boundaries originating from within the droplet itself^82^,
  1 $$G(x,y)=\exp \left(-\frac{{x}^{2}+{y}^{2}}{{\sigma }^{2}}\right),$$
  where *σ* denotes the width (standard deviation) of the bell-shaped function. For a very large value of *σ*, hardly any filtering is recorded. However, with decreasing the value of *σ*, the Gaussian filter starts to attenuate the high frequency components, removing those responsible for noise and other small scale (quickly varying) information. In our case, we have set the value of *σ* = 0.01 cycles per pixel (or one-hundredth of a wavelength per pixel) throughout this project. The pixel at the centre of the Fourier space receives the maximum weight, and the remaining coefficients drop off smoothly with increasing distance from the centre. Each image is filtered by multiplying its Fast Fourier Transform, i.e., ${{\boldsymbol{I}}}_{F}^{R}(\omega ,\nu )={\int }_{-\infty }^{+\infty }{\int }_{-\infty }^{+\infty }{{\boldsymbol{I}}}^{R}(x,y){e}^{-i(\omega x+\nu y)}{\rm{d}}x{\rm{d}}y$, with the Gaussian function defined in Eq. 1. Next, to access the spatial information of the filtered image, we perform the inverse Fourier Transform, i.e., ${{\boldsymbol{I}}}_{F}^{R}(x,y)=\frac{1}{2\pi }{\int }_{-\infty }^{+\infty }{\int }_{-\infty }^{+\infty }{{\bf{I}}}_{F}^{R}(\omega ,\nu ){e}^{i(\omega x+\nu y)}{\rm{d}}\omega {\rm{d}}\nu$. Figure 6d shows the cropped image of the red-channel blood droplet as a result of the filtering.
- Edge detection: Edges are common features in an image and are identified by rapid local variations in the image function. Edge detection is a process of identifying and locating sharp discontinuities in an image^83^. During the history of image processing, a variety of edge detection approaches have been devised which differ in their purpose and in their mathematical and algorithmical properties^84^. The location of edges in a 2D image are normally determined either by (i) finding the image gradient extrema (maximum or minimum) or by (ii) finding the zero-crossings of the Laplacian of the image. The Laplacian filter is a second derivative commonly used for edge detection and image enhancement in digital images. Second-order derivatives have a stronger response than first derivatives to fine detail, such as thin lines, weak edges and isolated points and that is due to the fact that first derivatives may not be large enough to distinguish the edge points, and, therefore, a weak edge can go undetected by such methods. Taking the second derivative of the points or Laplacian amplifies the changes in the first derivative and, therefore, increases the chances of detecting a weak edge^85^. Mathematically, the Laplacian of our 2D image ${{\boldsymbol{I}}}_{F}^{R}(x,y)$, is given by,
  2 $${\nabla }^{2}{{\boldsymbol{I}}}_{F}^{R}(x,y)=\left[\begin{array}{l}{{\boldsymbol{I}}}_{F}^{R}(x,y)\\ \\ {{\boldsymbol{I}}}_{F}^{R}(x,y)\end{array}\right]=\left[\begin{array}{l}\frac{{\partial }^{2}{{\boldsymbol{I}}}_{F}^{R}(x,y)}{\partial {x}^{2}}\\ \\ \frac{{\partial }^{2}{{\boldsymbol{I}}}_{F}^{R}(x,y)}{\partial {y}^{2}}\end{array}\right],$$
  and its magnitude, as shown in Fig. 6e, can be written as,
  3 $$\left\Vert {\nabla }^{2}{{\boldsymbol{I}}}_{F}^{R}(x,y)\right\Vert =\sqrt{{\left(\frac{{\partial }^{2}{{\boldsymbol{I}}}_{F}^{R}(x,y)}{\partial {x}^{2}}\right)}^{2}+{\left(\frac{{\partial }^{2}{{\boldsymbol{I}}}_{F}^{R}(x,y)}{\partial {y}^{2}}\right)}^{2}}.$$
- Polar Coordinate System: In this step, since the rich information in a circular dried blood droplet image is highly located in the radial or angular areas in reference to the centre point *r* = 0, we convert the magnitude of the image Laplacian, i.e., $\left\Vert {\nabla }^{2}{{\boldsymbol{I}}}_{F}^{R}(x,y)\right\Vert$, from Cartesian to Polar Coordinate System, i.e., $\left\Vert {\nabla }^{2}{{\boldsymbol{\Pi }}}_{F}^{R}(r,\theta )\right\Vert$ as shown in Fig. 6f. Using this technique is very important since it helps exhibit the symmetry of the pattern distribution in the blood droplet.
- Power spectrum: In the final pre-processing step prior to applying our machine learning algorithm, we apply the absolute value of the Fast Fourier Transform on the angular component of the logarithmic polar form of the image Laplacian of the red channel of each droplet image to calculate their power spectra, as illustrated in Fig. 6g. By applying the power spectrum on the angular component of the polar image the absolute measure of angle is removed, i.e., the remaining information becomes immune to rotations about the centre of the droplet. This extracts the spectral information and exhibits the pixel intensity within the spatial frequency domain for each image. This can be written as:
  4 $${{\boldsymbol{F}}}^{R}(\omega )=| {\rm{FFT}}({\rm{\log }}\,\parallel {\nabla }^{2}{{\boldsymbol{\Pi }}}_{F}^{R}(r,\theta )\parallel )| =\left|{\int }_{-\infty }^{+\infty }{\rm{\log }}\,\parallel \nabla {{\boldsymbol{\Pi }}}_{F}^{R}(r,\theta )\parallel {e}^{-i\omega \theta }{\rm{d}}\theta \right|.$$
  Furthermore, Fig. 6h shows that we take the logarithm of the power spectrum of the image, i.e., ${\Gamma }^{R}(\omega )={\rm{\log }}\,({F}^{R}(\omega ))$. This (i) enhances small pixel intensity and (ii) it works better than linear power spectra (see supplementary material).

### Machine learning algorithm

At this stage, our images are ready to be passed on to our machine learning algorithm for discrimination purposes. The input matrix **Γ**^*R*^ of each image is made of 48 rows and 103 columns, where each column represents the frequency of the spectral image, and each row represents radial component of the image. We have seen that the first row, i.e., *r* = 1, carries negligible radial information that could be useful in our subsequent steps. Similarly, the second half of the full frequency bandwidth, i.e, *c* ≥ 103/2 ≥ 52, exhibits minimal important features. Therefore, it is plausible to discard the first row and truncate the full frequency bandwidth to preserve only the first half, i.e., the first 52 columns. Hence, the input matrix **Γ**^*R*^ in this case consists of 47 rows and 52 columns (see supplementary material).

The input matrix **Γ**^*R*^ is originally a 4D matrix with the first dimension referring to the radial information within all the frequencies that make up the spectral image stacked up in a single column vector. The second, third and fourth dimensions represent the number of blood conditions, people and images taken from each person per condition, respectively, i.e., $\left[{{\boldsymbol{\Gamma }}}^{R}\right]=2444\times 5\times 30\times 12$. This matrix has been rearranged to be a 2D matrix after performing: (i) averaging over all, or some of, the images per volunteer (ii) normalisation of the image pixel intensities where minimum values are set to zero and maximum values are one, i.e., $\left[{{\boldsymbol{\Gamma }}}_{i}^{R}\right]=2444\times 30$, where *i* refers to the five blood conditions: baseline, peak, and after 2, 4, and 6 minutes.

This algorithm has three main steps to fully complete the discrimination process. It starts with an unsupervised learning that uses a dimensionality reduction method to extract the most varying components. For this step we use principal component analysis (PCA). Afterwards, a supervised learning method, linear discriminant analysis (LDA), is introduced which uses a linear combination of half of the largest PCA scores to find the vector that best separates the conditions of the blood images (see supplementary material). All the previous stages produce a machine learning algorithm that yields a fairly acceptable discrimination prediction outcome. However, the accuracy of this model can be significantly enhanced and the possibility of overfitting can be greatly lowered and hence the discrimination outcome will be noticeably improved by performing an optimising process which we shall describe next.

### Optimisation process

The novel idea of our optimisation process is a new strategy to find and use ’ideal’ training datasets that would best train the algorithm and result in the best overall discrimination, allowing the most predominant, generic discriminating features to be identified between the five experimental conditions. One approach to find these ’ideal’ data/participants is to systematically explore all possible combinations of 20, for example, out of 30 participants and compute the error rate for each individual iteration and the one yielding the combination of participants with the lowest error rate reveals the strongest generic discriminating features^86^. However, applying this method on our large image database would result in a total number of iterations of 30, 045, 015 which would take months to fully explore. Therefore, we have redesigned the structure of this optimisation process to be applicable to large datasets with less computational running times. Suppose the two blood condition matrices that we would like to optimise their discrimination are denoted by ***A*** and ***B***, then the optimisation method is constructed in two sets of iterations:

- First set of iterations: The main idea here is to find the ideal few participants in the first condition matrix ***A*** that best train our algorithm. Each iteration in this step builds a training dataset which is a concatenation of a ’selected’ slice of matrix ***A*** with matrix ***B***. This slice initially contains two participants from *A*: 1 and *i*, where *i* = 2, 29. At the end of each iteration tested on the validation dataset of ALL images in ***A*** and ***B***, the error rate is calculated as follows: each measurement (comprising of half of the largest PCA scores) is projected onto a two-dimensional LDA space, yielding a set of two LDA scores. In this space, the coordinates of the two centroids are calculated, and for each iteration, the Euclidean distances to both centroids are further calculated. The ratio of these two distances is used to assess whether the trial ends up in one of the blood conditions. The iterations ending up with the incorrect membership are expressed as a percentage error rate. The iteration that corresponds to the lowest error rate, *j*, is added to the following searching iterative process to the training slice of ***A***. Here we take 1,*j* and *i* which is the remaining 28 participants and the same process is repeated. After scanning all the possibilities with the lowest error rate, we now take participant 2 and *i* where *i* = 1, 3, …, 29 and the same iterative process is done again. By the end of these two main explorations, we choose the combinations that correspond to the lowest error rate and they will ultimately form the ideal choices to build the optimal training dataset of matrix ***A*** that corresponds to the first blood condition.
- Second set of iterations: The main idea here is to find the ideal participants in the second condition matrix ***B*** that best train our algorithm by using the ideal choices of ***A*** from the previous step. Taking the ideal training choices of the first blood condition, we perform the same iterative explorations on matrix ***B*** calculating the error rate values by the end of each iterations. Similarly to the first set of iterations, the lowest error rates obtained will build the optimal training dataset of matrix ***B***.

By the end of these two sets of iterations, we end up with two ideal training datasets corresponding to both blood conditions, i.e., ***A*** and ***B***. Passing these two training datasets on to our classifier highly enhances the discrimination outcome. It can be noticed that since we take only participants 1 and 2 from each matrix to search for the lowest error rate possibilities, this significantly decreases the optimisation time and accordingly enhances the overall discrimination process.

### Approval

The experimental protocols (exercise test and blood sampling) were approved by the Nottingham Trent University Human Ethical Review Committee.

### Accordance

The study conformed to the standard set by the Declaration of Helsinki.

### Informed consent

Informed consent was obtained from all participants/volunteers.

## Supplementary information

Supplementary Information.

## Data Availability

All relevant data are within the paper and its Supporting Information files. The dried blood images dataset generated and analysed during this study is available in the Figshare repository, 10.6084/m9.figshare.11373948.v1.
