# Supplementary material for: Machine Learning Analysis for Quantitative Discrimination of Dried Blood Droplets
Source: Sci Rep. 2020 Feb 24;10:3313. doi: 10.1038/s41598-020-59847-x (PMC7040018; doi:10.1038/s41598-020-59847-x)
Supplement: Supplementary file 1 — Supplementary Information. [file 41598_2020_59847_MOESM1_ESM.pdf]

Machine Learning Analysis for Quantitative Discrimination of Dried Blood Droplets

Supplementary Material

Lama Hamadeh[1], Samia Imran[1], Martin Bencsik[1], Graham Sharpe[1], Michael Johnson[1], and David J. Fairhurst[1]

[1] *Nottingham Trent University, Department of Physics and Mathematics, School of Science and Technology, Nottingham, Clifton Campus, NG11 8NS, United Kingdom*

December 19, 2019

Power Spectrum Matrix Cropping

We have seen in the paper that after obtaining the polar version of each droplet image of our dataset, we apply the absolute value of the Fast Fourier Transform on the angular component of the logarithmic polar form to calculate their power spectra. The resulting matrix, $\mathbf{\Gamma}^R(\omega)$, of each image is made of 48 rows and 103 columns, where each column represents the frequency of the spectral image, and each row represents radial component of the image, as shown in Fig1b. It can be seen that the first row, i.e., $r = 1$, carries negligible radial information that could be useful in our subsequent steps represented by a dark blue colour. Similarly, the second half of the full frequency bandwidth, i.e, $c \geq 103/2 \geq 52$, show minimal important features. Therefore, it is plausible to discard the first row and truncate the full frequency bandwidth to preserve only the first half, i.e., the first 52 columns. Hence, the input matrix $\mathbf{\Gamma}^R$ in this case is cut short and consists of 47 rows and 52 columns, as shown in Fig1c. This task not only preserves the important features needed for discrimination but also it decreases the computational time for subsequent discrimination steps.

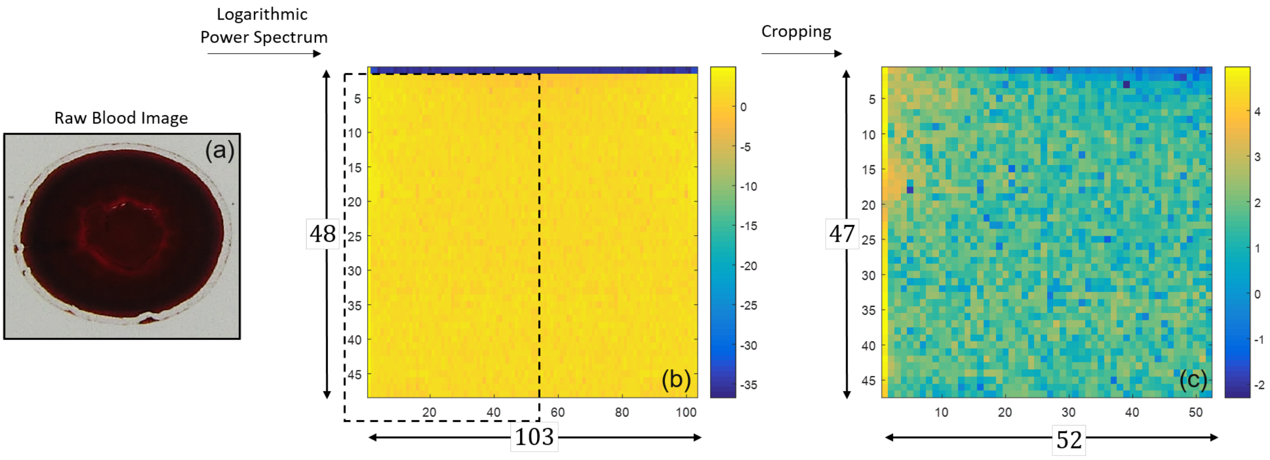

Figure 1: Frequency bandwidth cropping. (a) Shows the raw blood image. (b) Represents the full frequency bandwidth of the logarithmic power spectra of the image in (a) with 48 rows referring to the radial information and 103 columns referring to the frequencies. (c) Is the cropped logarithmic power spectrum with 48 rows and 52 columns.

Logarithm of the Power Spectrum

The high discrimination accuracy obtained by our machine learning algorithm would have not be achieved without the application of a sophisticated pre-processing image analysis. Specifically speaking, taking the logarithm of the power spectrum of each image of our database has not only enhanced the small pixel intensity but also, as we have found, it works better as a linear power spectra. Fig(2, a, b, d, e) show the power spectra of the averaged images as a function of the number of volunteers for two conditions; baseline and after 6 mins before and after applying the logarithmic power spectrum. The difference in both pairs of the spectra is quite clear. It can be noticed that applying the logarithm on the power spectrum enhances the final discrimination accuracy of our machine learning algorithm from 91.7% to 95%.

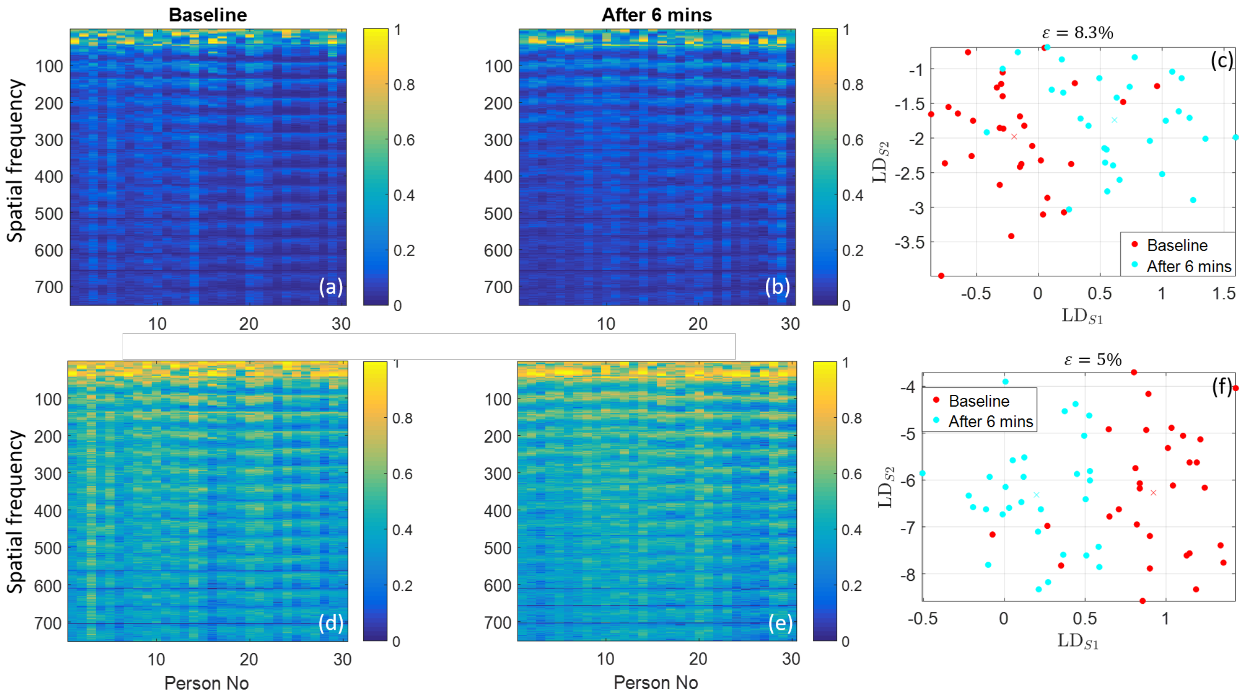

Figure 2: Power Spectrum vs logarithmic power spectrum. (a) and (b) represent the power spectrum of ”baseline” and ”after 6 mins” conditions corresponding to thirty volunteers. (c) shows the discrimination outcome using this power spectrum with an error rate of $\varepsilon = 8.3\%$. However, (d) and (e) exhibit the logarithmic power spectrum for the same conditions where the discrimination outcome, in (f), is enhanced with an error rate equal to $\varepsilon = 5\%$.

Machine Learning Algorithm

Our machine learning algorithm consists of three main steps: (i) dimensional reduction using Principal Component analysis (PCA), (ii) supervised classification using Linear Discriminant Analysis (LDA), and an optimisation process. Next, we will provide a brief overview of the two main methods used in this work (the optimisation process is clearly presented and explained in the paper).

Principal component analysis (PCA)

Principal component analysis is an unsupervised learning method, which seeks to find natural clustering of data in a lower dimensional subspace, i.e., feature space, whose basis contains linearly, ordered, independent, orthonormal and therefor uncorrelated vectors that correspond to the maximum-variance directions in the original space [4]. These vectors, also known as components, make up the columns of the covariance matrix and are eigenvectors, also referred to as loading vectors. The principal components (PCs) are ordered in terms of decreasing variance such that the majority of variation in the data can usually be described by the first couple of PCs and therefore the remaining PCs can be ignored reducing the dimensionality of the data [1]. However, depending on the research question this may not hold true and medium or lower ordered PCs may provide the necessary information rather than higher ordered PCs [5]. For any two blood conditions, computing the principal components using the Covariance Matrix Method that are associated to the largest variance usually follows generic steps. Given the logarithmic power spectrum database for each condition, $\boldsymbol{\Gamma}_i^R$, with the size of 2444×30, we build the training dataset which is a concatenation of a slice from the first condition's matrix and the total second condition's matrix. Then, we calculate the mean of all the training dataset samples followed by centering this dataset, i.e., subtract the mean from each sample in the dataset. Once the covariance matrix is constructed, i.e., $\boldsymbol{C} = \boldsymbol{\Gamma}_i^R \times \left(\boldsymbol{\Gamma}_i^R\right)^T$, we diagonalise it to obtain its eigenvectors and eigenvalues. The principal components, or the so-called scores, are calculated by projecting the centred training dataset on the eigenvectors that correspond to the largest eigenvalues. These scores formulate the feature space, a sub-space of the original space containing the highest variations in the data needed for subsequent supervised discrimination process.

Linear discriminant analysis (LDA)

In general terms, the discrimination process uses the information in a learning dataset of labeled observations to construct a classifier that will separate the predefined classes as much as possible. Discriminating factors of interest to the user may go undetected if they are of small magnitude, and/or if they end up being shared between too many principal components. Thus, to identify subtle discrimination features that exist between the blood conditions, a supervised algorithm such as linear discriminant analysis (LDA) is required [1]. Despite the plethora of classifier techniques, we have chosen LDA for its simplicity and well-established behaviour. The idea of the LDA was first proposed by Fisher [2] in the context of taxonomy. Mathematically speaking, LDA attempts to find a set of directions in feature space, i.e., decision space, where both classes are well-separated when projected onto the chosen sub-space. Thus, the goal of the LDA is to find a suitable projection that maximises the distance between the inter-class data while minimising the intra-class data [3]. For a two-class LDA, the above idea results after taking into account certain percentage of the PCA scores that we have obtained in the previous step as a learning dataset. Next, we construct a projection $\boldsymbol{w}$ that can be formulated in an equation that is commonly known as the generalised Rayleigh quotient and it is given by[3]:

$$\boldsymbol{w} = \arg \max_{\boldsymbol{w}} \frac{\boldsymbol{w}^T \boldsymbol{S}_{\mathrm{B}} \boldsymbol{w}}{\boldsymbol{w}^T \boldsymbol{S}_{\mathrm{W}} \boldsymbol{w}}, \tag{1}$$

where the scatter matrices for between-class $\boldsymbol{S}_{\mathrm{B}}$ and within-class $\boldsymbol{S}_{\mathrm{W}}$ data are given by:

$$\boldsymbol{S}_{\mathrm{B}} = (\mu_2 - \mu_1)(\mu_2 - \mu_1)^T, \tag{2a}$$

$$\boldsymbol{S}_{\mathrm{W}} = \sum_{j=1}^{2} \sum_{x} (\boldsymbol{x} - \mu_j)(\boldsymbol{x} - \mu_j)^T, \tag{2b}$$

where: μ_1 and μ_2 are the mean values for the first and second classes, and $\boldsymbol{x}$ is the selected maximum-variance PCA scores matrix. These quantities essentially measure the variance of the datasets as well as the variance of the differences in the means [3]. Once these matrices are constructed, diagonalising Eq.1 becomes possible where the maximum eigenvalue and its associated eigenvector give the quantity of interest and the projection basis. To obtain the LDA scores, the first two in our case, we project the validation matrix, which is a concatenation of all the data in the logarithmic power spectrum matrices for both blood conditions, on the resulting LDA eigenfunctions. Essentially, these scores show the 'best' discrimination the logarithmic power spectrum of both blood conditions could achieve.

References

[1] M. Bisele et al. “Optimisation of a machine learning algorithm in human locomotion using principal component and discriminant function analyses”. In: *PLOS ONE* 12 (2017), pp. 1–19.

[2] R. A. Fisher. “THE USE OF MULTIPLE MEASUREMENTS IN TAXONOMIC PROBLEMS”. In: *Wiley Online Library* 7 (2 1936), pp. 179–188.

[3] J. N. Kutz. *Data-Driven Modeling and Scientific Computation: Methods for Complex Systems and Big Data*. Oxford University Press, 2013.

[4] A.M. Martinez and A.C. Kak. “PCA versus LDA”. In: *IEEE Transactions on Pattern Analysis and Machine Intelligence* 23 (2 2001), pp. 228–233.

[5] A. Phinyomark et al. “Do intermediate-and higher-order principal components contain useful information to detect subtle changes in lower extremity biomechanics during running?” In: *Human Movement Science* 44 (2015), pp. 91–101.
